# Influence of In Utero Maternal and Neonate Factors on Cord Blood Leukocyte Telomere Length: Clues to the Racial Disparity in Prostate Cancer?

**DOI:** 10.1155/2016/3691650

**Published:** 2016-12-14

**Authors:** Kari A. Weber, Christopher M. Heaphy, Sabine Rohrmann, Beverly Gonzalez, Jessica L. Bienstock, Tanya Agurs-Collins, Elizabeth A. Platz, Alan K. Meeker

**Affiliations:** ^1^Department of Epidemiology, Johns Hopkins Bloomberg School of Public Health, 615 N. Wolfe Street, Baltimore, MD 21205, USA; ^2^Department of Pathology, Johns Hopkins University School of Medicine, 600 N. Wolfe Street, Baltimore, MD 21287, USA; ^3^Sidney Kimmel Comprehensive Cancer Center at Johns Hopkins, 401 N. Broadway, Baltimore, MD 21287, USA; ^4^Department of Cancer Epidemiology and Prevention, Institute of Social and Preventive Medicine, University of Zurich, Hirschengraben 84, 8001 Zurich, Switzerland; ^5^Department of Gynecology and Obstetrics, Johns Hopkins University School of Medicine, 600 N. Wolfe Street, Baltimore, MD 21287, USA; ^6^Division of Cancer Control and Population Sciences, National Cancer Institute, 9609 Medical Center Drive, Bethesda, MD 20892, USA; ^7^Department of Urology and the James Buchanan Brady Urological Institute, Johns Hopkins University School of Medicine, 600 N. Wolfe Street, Baltimore, MD 21287, USA

## Abstract

*Background*. Modifiable factors in adulthood that explain the racial disparity in prostate cancer have not been identified. Because racial differences in utero that may account for this disparity are understudied, we investigated the association of maternal and neonate factors with cord blood telomere length, as a cumulative marker of cell proliferation and oxidative damage, by race. Further, we evaluated whether cord blood telomere length differs by race.* Methods*. We measured venous umbilical cord blood leukocyte relative telomere length by qPCR in 38 black and 38 white full-term male neonates. Using linear regression, we estimated geometric mean relative telomere length and tested for differences by race.* Results*. Black mothers were younger and had higher parity and black neonates had lower birth and placental weights. These factors were not associated with relative telomere length, even after adjusting for or stratifying by race. Relative telomere length in black (2.72) and white (2.73) neonates did not differ, even after adjusting for maternal or neonate factors (all *p* > 0.9).* Conclusions*. Maternal and neonate factors were not associated with cord blood telomere length, and telomere length did not differ by race. These findings suggest that telomere length at birth does not explain the prostate cancer racial disparity.

## 1. Introduction

The racial disparity in prostate cancer incidence and mortality rates is among the greatest across all cancer sites. US black men have a 60% higher risk of prostate cancer and greater than twice the risk of dying of prostate cancer [1]. Further, black men tend to fair worse following surgical intervention of their primary prostate cancer and are more likely to die of their prostate cancer compared with white men [2–4]. Despite extensive study, modifiable factors measured in adulthood that may explain this disparity have not been found [5, 6]. However, early life exposures have not been systematically studied as an explanation for this racial disparity [7].

One potential early life mechanism that could influence the racial disparity in prostate cancer is fetal programming. The “fetal origin hypothesis” states that exposures in utero may “program” a fetus, that is, permanently change its structure and metabolism, resulting in altered chronic disease risk later in life [8, 9]. If the prevalence or extent of such exposures differs by race, then the degree of fetal programming could account for racial differences in chronic diseases (e.g., prostate cancer).

Previous work suggests that birth characteristics, as indicators of the fetal environment, are associated with later life risk of prostate cancer [10–15]. For example, positive associations [10, 11] and suggestive positive associations [12–14] have been found between higher birth weight and prostate cancer, especially advanced-stage disease and mortality in some studies, but not in others [15]. Associations between other birth characteristics and aggressive prostate cancer have been reported including longer length at birth and metastatic prostate cancer [14] and ponderal index and death from prostate cancer [15]. There was also a suggestive association between higher placental weight and death from prostate cancer in one study [15]. Additional studies have indirectly addressed the influence of the in utero environment on the prostate cancer racial disparity. For example, we previously reported slightly higher concentrations of testosterone, estradiol [16], and leptin [17] and slightly lower concentrations of insulin-like growth factors [16] and vitamin D [18] in the cord blood of black male neonates compared with white male neonates. In addition, higher testosterone and androstenedione concentrations in black compared to white mothers have been previously reported at the beginning of gestation [19, 20] or at time of delivery [21]. These findings suggest that risk factors previously investigated for prostate cancer later in life may differ in utero by race. However, these markers reflect differences at specific points during gestation, rather than the effect of differences in cumulative exposure in utero. Thus, markers of the cumulative influence of the in utero milieu on the fetus are needed.

Telomeres, repetitive DNA sequences that protect the ends of the chromosomes, may be such a marker. Telomere length is heritable, but for any given cell, telomeres shorten with each round of cell replication and with exposure to oxidative damage [22–24]. Therefore, telomere length in leukocytes in cord blood may reflect an individual's starting point at birth, possibly integrating across cumulative proliferation and oxidative exposures during gestation. Supporting this contention, a few recent studies have observed associations of lower maternal folate intake and greater maternal stress with shorter neonate telomere length [25, 26]. Further, telomere length is an attractive marker beyond reflecting cumulative exposure because telomere shortening contributes to carcinogenesis, including prostate cancer [27–30]. Thus, telomere shortening may directly link inheritance, cumulative in utero exposures, and prostate cancer. Here, we investigated the associations between race, maternal and neonate factors, and cord blood leukocyte telomere length in male neonates. An abstract on this work was previously published [31].

## 2. Methods

### 2.1. Study Population and Assessment of Maternal and Neonate Factors

The hormones in umbilical cord blood study (HUB) was a pilot study conducted by the Sidney Kimmel Comprehensive Cancer Center at Johns Hopkins and the Howard University Cancer Center Partnership and approved by the Institutional Review Boards of the Prince George's Hospital Center and the Johns Hopkins Bloomberg School of Public Health. In 2004-2005, venous umbilical cord blood samples (*N* = 240) were collected from eligible neonates from the Johns Hopkins Hospital in Baltimore, MD, and the Prince George's Hospital Center in Cheverly, MD. The eligibility criteria included singleton, full-term birth (37–42 weeks), normal range birth weight (2500–4000 g), black or white, no pregnancy complications, and no maternal use of hormonal medications during pregnancy. Nurses completed a standardized study form on maternal race, age, and parity (maternal factors), birth and placental weights, and time of birth (neonate factors). At delivery, nurses drew 15 mL samples of venous umbilical cord blood into 2 tubes containing sodium EDTA. As previously described, samples were stored in a refrigerator and processed, usually within 12 hours, into plasma, buffy coat, and red cells; aliquots were stored at −70°C in cryovials [16]. Cord blood concentrations of steroid and peptide hormones, which reflect both maternal and neonate contributions, were previously measured for testosterone, androstanediol glucuronide (AAG), estradiol, sex hormone binding globulin (SHBG), insulin-like growth factor (IGF) axis (IGF-1, IGF-2, IGF binding protein [IGFBP-3]) [16], 25-hydroxyvitamin D [18], and leptin [17]. Bioavailable testosterone and estradiol were estimated as the molar ratio of each to SHBG [16]. For this study, we used the 76 male samples collected at Johns Hopkins (38 white and 38 black).

### 2.2. Cord Blood Leukocyte Relative Telomere Length Determination

Leukocyte DNA was isolated using the DNeasy Blood and Tissue kit (Qiagen, Venlo, Netherlands). Quantitative PCR was used to estimate the ratio of telomeric DNA to that of a single copy gene (*β*-globin) [32], with the following modifications [33]. Briefly, 5 ng of genomic DNA was used in a 25 *μ*L volume for either the telomere or *β*-globin reactions; each sample was run in triplicate. Each 96-well plate contained a no template negative control and two separate 5-point standard curves using leukocyte DNA; these standard curves allowed the PCR efficiency to be determined for each experimental run. Each plate also included three samples isolated from a series of cell lines with known telomere lengths, ranging from 3–15 kb, as determined independently by telomere restriction fragment analysis. Inclusion of these samples provided an additional quality control check. The mean telomere threshold (*C*_*t*_) value and the *β*-globin *C*_*t*_ value were calculated from the telomere and the *β*-globin triplicate reactions, respectively. For each sample, the telomere of the experimental sample to the single copy gene (T/S) ratio (−*dC*_*t*_) was calculated by subtracting the *β*-globin *C*_*t*_ value from the telomere *C*_*t*_ value. The relative ratio (−*ddC*_*t*_) was determined by subtracting the −*dC*_*t*_ from a 5 ng sample in the cell line series from the −*dC*_*t*_ of each unknown sample. Across all samples, the mean CV was 0.96% and 1.00% (maximum CVs were 4.38% and 2.70%) for the telomere and *β*-globin reactions, respectively.

### 2.3. Statistical Analysis

Means of maternal and neonate factors were calculated by race and differences were assessed using *t*-tests. Using linear regression, we estimated geometric mean relative telomere length and 95% confidence intervals (CI) by the maternal and neonate factors and the cord blood steroid and peptide hormones, overall, after adjusting for race, and then stratified by race. We estimated the relative change in geometric mean relative telomere length per unit or per standard deviation of each maternal and neonate factor and tested for trend and interaction by race using the Wald test. Finally, we estimated geometric mean relative telomere length by race, overall and after adjusting for the maternal and neonate factors and for the cord blood steroid and peptide hormones. All analyses were performed using SAS 9.3 (SAS Institute, Cary, NC).

## 3. Results

The differences in maternal and neonate factors by race are shown in Table 1. Compared to white mothers, black mothers were younger (29 years versus 24 years; *p* < 0.005) and had higher parity (0.6 versus 1.3; *p* = 0.03). Compared to white neonates, black neonates had lower birth (3470.4 g versus 3207.2 g; *p* = 0.004) and placental weights (701.7 g versus 640.9 g; *p* = 0.05).  Table 2 shows geometric mean relative telomere length in umbilical cord blood leukocytes by quartiles of maternal and neonate factors, overall, adjusting for race, and stratified by race. None of these maternal or neonate factors were associated with relative telomere length before or after adjusting for race or when stratifying by race (all *p*-trend > 0.4).

Relative change in geometric mean leukocyte relative telomere length per standard deviation change is shown for steroid and peptide hormone concentrations (Table 3). For the majority of the steroid and peptide hormones, we did not observe a statistically significant change in relation to the geometric mean leukocyte telomere length (all *p*-trend > 0.1). However, for androstanediol glucuronide (AAG), relative telomere length was 11% longer per standard deviation (15.11 ng/mL) increase in AAG (*p*-trend = 0.01) and remained statistically significant after adjusting for race (*p*-trend = 0.01). After stratifying by race, the association between AAG and relative telomere length was similar in magnitude in black and white neonates (*p*-interaction = 0.9) but was statistically significant only in black neonates (*p*-trend = 0.01).

Finally, as shown in Table 4, cord blood leukocyte relative telomere length did not differ between black (2.72, 95% CI 2.44–3.04) and white (2.73, 95% CI 2.45–3.05) male neonates. These results were unchanged after adjusting for maternal factors (age and parity), neonate factors (birth weight and placental weight), or the steroid and peptide hormones (data not shown).

## 4. Discussion

To address the role of early life factors that may contribute to the racial disparity of prostate cancer, we explored the association between maternal (mother's age and parity) and neonate (birth and placental weights) factors with relative telomere length, a marker of the cumulative influence of the prenatal environment, in black and white male neonates. We hypothesized that maternal and neonate factors would be associated with relative telomere length and would be a possible measure of differences in fetal programming between black and white neonates. Given the racial disparity in prostate cancer and our prior work on telomere length and prostate cancer risk and outcomes [1, 27, 28, 30], we hypothesized that relative telomere length would be shorter in black than white neonates. However, these maternal and neonate factors were not associated with cord blood leukocyte relative telomere length, and cord blood leukocyte relative telomere length did not differ between black and white male neonates.

While maternal age, parity, and birth weight were associated with race, none of the maternal or neonate factors were associated with relative telomere length. However, we did observe a positive association between AAG and relative telomere length. That association was present overall and within black neonates, although in our prior work AAG concentrations did not differ by race [16]. Few prior studies in humans have assessed parental and neonate determinants of newborn leukocyte telomere length. The most consistent findings to date are that older paternal age [34] and greater maternal psychosocial stress [26] are associated with shorter newborn leukocyte telomere length. Two recent studies have also observed associations between decreased maternal folate concentrations [25] and being large for gestational age [35] with shorter neonate telomere length. Some studies report that adverse pregnancy complications, such as gestational diabetes and preeclampsia, are associated with shorter newborn telomere length [36]. Murine studies showing the prenatal determinants of neonate telomere lengths are not available to the best of our knowledge since inbred mouse strains have very long telomere lengths and thus are not directly comparable to human telomere lengths [37].

Two previous studies also assessed racial differences in neonate telomere length with conflicting results [38, 39]. Our findings were consistent with Okuda et al. (*n* = 134), finding no difference between cord blood leukocyte telomere length in black and white neonates, although both males and females were included in their study [38]. Drury et al. (*n* = 66), also including both males and females, found black neonates to have significantly longer telomeres than white neonates and black females to have the longest [39]. There may be differences between these studies and ours due to differences in the array of potential confounders, specifically sex of the neonate. If black females have significantly longer telomeres than white males, white females, and black males, then these differences may affect the overall difference in telomere lengths between black and white neonates. Furthermore, all three studies also have small sample sizes and thus chance cannot be ruled out as a cause of the differences in results.

Although cord blood leukocyte relative telomere length did not differ between black and white neonates in our study, our observations do not rule out early postnatal racial differences in telomere length. The black neonates were smaller on average than the white neonates, as is seen nationally [40]. It is possible that since black neonates, on average, begin life smaller, they may experience compensatory catch-up growth that may, in turn, affect postnatal telomere lengths. In keeping with this idea, a longitudinal study found that black, low-birth weight neonates experienced greater catch-up growth and were close to the standard weight by age two while the white neonates were not standard weight [41]. Rapid catch-up growth has been shown to affect programming and increase the risk of diseases such as cardiovascular disease and diabetes later in life [42] and obesity later in life [43]. Some animal models have also shown that rapid postnatal growth following low-protein gestation was associated with accelerated telomere shortening in their aorta, pancreatic islets, and renal tissues [44]. A recent longitudinal study measuring telomere length at birth and midlife showed that longer telomere length at birth was associated with a greater decrease in telomere length in adulthood overall [45]. In that study, black neonates had longer telomere lengths at birth and therefore, greater telomere shortening to adulthood compared with whites; this relationship remained after adjustment for parental income and educational attainment. However, after stratification by gender, no association was present among males, even after adjustment for parental factors [45]. Our findings are consistent with those observations. However, the overall findings from that longitudinal study suggest that the combination of compensatory catch-up growth and greater telomere shortening may account for some of the racial disparity in later life chronic diseases, such as prostate cancer.

Some aspects of our study warrant discussion. The HUB Study was designed specifically to investigate differences in the in utero environment that may account for racial disparities in prostate cancer later in life. As such, the race of the parents and neonate were documented to be the same. Due to the eligibility criteria, none of the mothers had pregnancy complications and all neonates were full-term with normal birth weight and thus, relevant to normal pregnancies rather than to extreme pregnancy settings. We did not collect paternal information; father's characteristics may also influence the gestational environmental [34]. While our study was designed to investigate racial differences in telomere length that may account for racial disparities in prostate cancer later in life, we cannot directly measure who will and will not develop prostate cancer due to the feasibility of follow-up from birth to average age at diagnosis of prostate cancer later in adulthood. However, since telomere length is a marker of the in utero environment, our findings may inform studies on other types of cancer and other diseases, particularly those for which existing epidemiologic data indicates potential associations with telomere length.

The steroid and peptide cord blood biomarkers of the in utero environment were previously measured, thus making our study efficient. In addition, our study is one of the first to evaluate associations of these biomarkers in the in utero environment with telomere length in cord blood leukocyte DNA at time of birth.

We did not measure the change in relative telomere length across gestation in the neonate for feasibility reasons; thus we do not know if the rate of telomere shortening during gestation is the same by race. We could not feasibly measure telomere length in the immature prostate, the target organ, of the neonates, and while we do not know the correlation between neonate peripheral blood leukocyte telomere length and neonate prostate cell telomere length, published studies indicate a strong correlation between telomere length of different somatic tissues and leukocytes in adults [46] and between different fetal tissues [47]. The lack of association of maternal and neonate factors with relative telomere length may have resulted from the use of crude measures of this environment. For example, we measured placental weight, but not other placental characteristics, such as width and shape, that may better capture the placenta's efficiency in transporting nutrients and oxygen from the mother to the fetus. Placental shape, but not placental weight, was associated with an increased risk of colorectal cancer [48], and small or large placental surface area was associated with an increased risk of lung cancer [49] in the Helsinki Birth Cohort studies. Finally, although our study sample size was small, it was powered to detect a minimum difference of 0.23 on the log scale. We hypothesized that the difference in geometric mean relative telomere length between black and white neonates would need to be large to account for the large disparity in prostate cancer risk (60% higher incidence).

## 5. Conclusions

In conclusion, these findings do not support telomere length at birth as a marker for differences in fetal programming among black neonates compared to white neonates and do not appear to explain the racial disparity in prostate cancer later in life. Future investigations utilizing larger sample sizes, potentially including other lower risk racial groups like Asian/Pacific Islander [1] for comparison, with more maternal information and the addition of paternal information, may help to fully elucidate possible inherent and in utero influences on leukocyte telomere length and consequences for the racial disparities in prostate cancer and other health states observed later in life.

## Figures and Tables

**Table 1:** Maternal and neonate factors by race, males in the hormones in umbilical cord blood study.

	Black	White	*p* value
*N*	38	38	
Maternal factors			
Mean age (years)	24	29	0.005
Mean parity	1.3	0.6	0.03
Neonate factors			
Mean birth weight (g)	3,207.2	3,470.4	0.004
Mean placental weight (g)	640.9	701.7	0.05

**Table 2:** Geometric mean relative telomere length in umbilical cord blood leukocytes by maternal and neonate factors, overall and by race, males in the hormones in umbilical cord blood study.

	Geometric mean (95% CI)			
	Unadjusted	Adjusted for race	Black	White
*Maternal factors*				
Age (years)				
≤19	2.60 (2.21–3.05)	2.58 (2.19–3.05)	2.70 (2.26–3.23)	2.35 (1.67–3.30)
20–25	2.72 (2.29–3.22)	2.70 (2.26–3.22)	2.73 (2.27–3.29)	2.67 (1.83–3.90)
26–31	3.01 (2.59–3.49)	3.03 (2.59–3.53)	3.23 (2.48–4.20)	2.93 (2.41–3.56)
≥32	2.58 (2.23–3.00)	2.59 (2.23–3.02)	2.37 (1.86–3.03)	2.70 (2.20–3.30)
Relative change per year	1.00	1.00	1.00	1.01
*p-*trend	*0.9*	*1.0*	*0.6*	*0.6*

Parity				
0	2.65 (2.38–2.96)	2.65 (2.37–2.96)	2.61 (2.20–3.11)	2.68 (2.30–3.11)
1	2.96 (2.52–3.48)	2.96 (2.52–3.48)	2.77 (2.24–3.42)	3.22 (2.48–4.18)
2	2.82 (2.27–3.50)	2.82 (2.27–3.51)	2.99 (2.28–3.93)	2.58 (1.78–3.73)
≥3	2.52 (2.00–3.16)	2.52 (2.00–3.19)	2.67 (2.07–3.44)	2.05 (1.21–3.46)
Relative change per birth	0.99	0.99	1.00	0.98
*p-*trend	*0.8*	*0.8*	*1.0*	*0.7*

*Neonate factors*				
Birth weight (g)				
≤3025	2.68 (2.30–3.14)	2.67 (2.28–3.13)	2.76 (2.29–3.32)	2.56 (1.96–3.35)
3026–3373	2.52 (2.16–2.95)	2.51 (2.14–2.95)	2.58 (2.16–3.09)	2.40 (1.80–3.21)
3374–3634	3.01 (2.58–3.52)	3.02 (2.58–3.54)	2.49 (1.95–3.18)	3.36 (2.74–4.13)
≥3635	2.71 (2.32–3.17)	2.73 (2.32–3.20)	3.29 (2.53–4.29)	2.48 (2.04–3.02)
Relative change per SD (399.99)	1.00	1.00	1.00	1.00
*p-*trend	*0.9*	*1.0*	*1.0*	*0.9*

Placental weight (g)				
≤585	2.48 (2.12–2.90)	2.48 (2.12–2.90)	2.52 (2.06–3.09)	2.42 (1.85–3.16)
586–659	2.90 (2.48–3.40)	2.90 (2.48–3.40)	2.76 (2.23–3.41)	3.07 (2.39–3.95)
660–760	2.77 (2.37–3.24)	2.77 (2.37–3.25)	2.84 (2.33–3.48)	2.67 (2.05–3.49)
≥761	2.77 (2.37–3.24)	2.77 (2.36–3.25)	2.81 (2.14–3.69)	2.75 (2.23–3.39)
Relative change per SD (138.01)	1.02	1.02	1.06	1.00
*p-*trend	*0.6*	*0.6*	*0.4*	*1.0*

**Table 3:** Relative change in the geometric mean umbilical cord blood leukocyte telomere length per standard deviation change in umbilical cord blood hormone concentrations, overall and by race, males in the hormones in umbilical cord blood study.

Hormone	Standard deviation	Unadjusted		Adjusted for race		Black		White	
		Relative change	*p-*trend	Relative change	*p-*trend	Relative change	*p-*trend	Relative change	*p-*trend
Testosterone (ng/mL)	0.47	0.99	0.7	0.99	0.7	0.99	0.7	0.99	0.9
Estradiol (pg/mL)	5,464	0.97	0.4	0.97	0.4	0.98	0.8	0.96	0.5
SHBG (nmol/L)	5.00	0.97	0.4	0.97	0.4	0.93	0.1	1.01	0.9
Androstanediol glucuronide (ng/mL)	15.11	1.11	**0.01**	1.11	**0.01**	1.12	**0.01**	1.08	0.3
IGF-1 (ng/mL)	51.76	1.02	0.6	1.02	0.6	1.05	0.6	1.02	0.8
IGF-2 (ng/mL)	93.43	1.03	0.4	1.04	0.4	1.09	0.1	1.00	0.9
IGFBP-3 (ng/mL)	394.8	0.99	0.7	0.98	0.7	0.99	0.9	0.98	0.7
Leptin (pg/mL)	8,897	1.00	0.9	1.00	0.9	1.15	0.3	0.99	0.8
Vitamin D (ng/mL)	9.45	0.97	0.4	0.96	0.3	1.01	0.9	0.93	0.2
Molar ratios of									
Testosterone/SHBG	0.13	1.03	0.5	1.03	0.5	1.04	0.4	1.01	0.9
Estradiol/SHBG	1.09	1.00	0.9	0.99	0.9	1.05	0.5	0.97	0.6
IGF-1/IGFBP-3	0.08	1.06	0.1	1.07	0.1	1.05	0.4	1.08	0.2
IGF-2/IGFBP-3	0.19	1.06	0.1	1.06	0.1	1.09	0.08	1.02	0.7

**Table 4:** Geometric mean telomere length in umbilical cord blood leukocytes by race, males in the hormones in umbilical cord blood study.

	Geometric mean relative telomere length (95% CI)		
	Black	White	*p* value
Unadjusted	2.72 (2.44–3.04)	2.73 (2.45–3.05)	0.96
Adjusted for maternal factors^*∗*^	2.74 (2.43–3.09)	2.72 (2.41–3.07)	0.93
Adjusted for neonate factors^*∗∗*^	2.73 (2.43–3.06)	2.73 (2.43–3.06)	0.99

^*∗*^Mother's age and parity.

^*∗∗*^Birth weight and placental weight.
